# Financial toxicity in cancer patients and subsequent risk of repeat acute care utilization

**DOI:** 10.3389/fpsyg.2023.1209526

**Published:** 2023-08-17

**Authors:** Julia J. Shi, J. Alberto Maldonado, Chi-Fang Wu, Susan K. Peterson, Ying-Shiuan Chen, Kevin Diao, Robert J. Volk, Sharon H. Giordano, Ya-Chen T. Shih, Kelsey Kaiser, Grace L. Smith

**Affiliations:** ^1^Department of Radiation Oncology, The University of Texas MD Anderson Cancer Center, Houston, TX, United States; ^2^John Sealy School of Medicine, The University of Texas Medical Branch, Galveston, TX, United States; ^3^Department of Health Services Research, The University of Texas MD Anderson Cancer Center, Houston, TX, United States; ^4^Department of Behavioral Science, The University of Texas MD Anderson Cancer Center, Houston, TX, United States

**Keywords:** financial toxicity, cancer, acute care, emergency department, screening

## Abstract

**Background:**

Acute care (AC) visits by cancer patients are costly sources of healthcare resources and can exert a financial burden of oncology care both for individuals with cancer and healthcare systems. We sought to identify whether cancer patients who reported more severe initial financial toxicity (FT) burdens shouldered excess risks for acute care utilization.

**Methods:**

In 225 adult patients who participated in the Economic Strain and Resilience in Cancer (ENRICh) survey study of individuals receiving ambulatory cancer care between March and September 2019, we measured the baseline FT (a multidimensional score of 0–10 indicating the least to most severe global, material, and coping FT burdens). All AC visits, including emergency department (ED) and unplanned hospital admissions, within 1-year follow-up were identified. The association between the severity of FT and the total number of AC visits was tested using Poisson regression models.

**Results:**

A total of 18.6% (*n* = 42) of patients had any AC visit, comprising 64.3% hospital admissions and 35.7% ED visits. Global FT burden was associated with the risk of repeat AC visits within 1-year follow-up (RR = 1.17, 95% CI 1.07–1.29, *P* < 0.001 for every unit increase), even after adjusting for sociodemographic and disease covariates. When examining subdimensions of FT, the burden of depleted FT coping resources (coping FT) was strongly associated with the risk of repeat AC visits (RR = 1.27, 95% CI 1.15–1.40, *P* < 0.001) while material FT burden showed a trend toward association (RR = 1.07, 95% CI 0.99–1.15, *P* = 0.07).

**Conclusion:**

In this prospective study of acute oncology care utilization outcomes among adult cancer patients, FT was a predictor of a higher burden of acute care visits. Patients with severely depleted material and also practical and social coping resources were at particular risk for repeated visits. Future studies are needed to identify whether early FT screening and intervention efforts may help to mitigate urgent acute care utilization burdens.

## Introduction

Cancer patients and survivors experience significant personal economic burdens from direct out-of-pocket medical costs, productivity losses, and employment disability. These burdens can total up to thousands of dollars of financial burden to the patient annually, ultimately leading to financial toxicity (FT) after the diagnosis and treatment of the disease in up to half of individuals with cancer (Pisu et al., 2018; Smith et al., 2019; Mariotto et al., 2020). FT disproportionately impacts vulnerable cancer patients—those who are younger or socioeconomically disadvantaged (Pisu et al., 2018; Smith et al., 2019) and the financial burdens can be exacerbated when the patients require acute care through repeated emergency department (ED) visits and unplanned hospitalizations (Peery et al., 2015; Albright et al., 2019; Whitney et al., 2019).

In general populations of elderly patients, evidence suggests that individuals reporting more severe health-related social needs—such as financial, food, transportation, or housing insecurity as well as loneliness—subsequently require more frequent ED and acute hospital visits, including avoidable causes. Avoidable causes in these general patient populations include infection, exacerbation of chronic cardiopulmonary conditions, and falls or trauma. Such prior evidence has therefore prompted and supported the rationale for enhancing social needs screening and early intervention on health-related social needs in general medical settings to help mitigate costly acute care utilization burdens and associated poor health outcomes in the general population (Oh et al., 2018; Lash et al., 2022; Alishahi Tabriz et al., 2023).

In cancer patients, however, it remains unclear whether a similar association exists for health-related social needs predicting excess acute care utilization in this population. Such evidence would support FT interventions as a potential tool for mitigating costly and repeated acute care utilization specifically for this group (Traeger et al., 2015; Basch et al., 2017; Nipp et al., 2019). To address this knowledge gap, the evaluation of acute care utilization patterns associated with cancer-related FT is needed. Cancer-related FT is a construct that incorporates the needs that arise from both health-related material and psychosocial coping burdens. Furthermore, FT can be captured using validated measurement in individuals with cancer (Smith et al., 2019, 2021, 2022; Blinder et al., 2022). Thus, the excess risks for both avoidable and unavoidable acute care utilization associated with FT in the oncology care setting can be quantitatively elucidated. For cancer populations, avoidable causes of acute care utilization could include anemia, nausea, vomiting and dehydration, fever, and infection (Alishahi Tabriz et al., 2023). Such an analysis in cancer patients is important to inform current efforts to expand FT screening (Bradley et al., 2021; Shih et al., 2022), define relevant adverse outcomes associated with FT, and create intervention strategies to mitigate adverse outcomes in this population (Smith et al., 2022). Therefore, to advance this understanding, we conducted a survey-based study to quantify FT in a diverse sample of adult patients with cancer and prospectively characterized acute healthcare utilization patterns. The primary objective of this analysis was to quantify the association between the severity of patient-reported FT and subsequent acute care utilization within the following year. We hypothesized that patients experiencing more severe FT would experience a higher burden of acute care utilization.

## Methods

This study was approved by the University of Texas M. D. Anderson Cancer Center Institutional Review Board.

### Data sources and patient sample

Eligible participants were enrolled in the Economic Strain and Resilience in Cancer (ENRICh) study (the parent study was previously reported in prior publications Smith et al., 2021; Xu et al., 2022) between March 2019 and September 2019. All participants were at least 18 years old, receiving ambulatory cancer care for pathologically confirmed cancer in 1 of 14 different radiation, surgical, or medical oncology clinics at a comprehensive cancer center main campus or community-based satellite clinical sites. Of the 364 patients approached for study participation, 232 patients (64%) agreed to participate. Excluded from analysis were patients who did not answer at least half of the survey questions (*N* = 1), did not consent to medical record review (*N* = 2), or were lost to clinical follow-up after the survey date (*N* = 4), leaving a final analytic sample size of *N* = 225.

### Outcome: acute care visits

We extracted from the electronic medical record encounters for any unplanned hospitalizations or emergency care visits in the oncology center, which were defined as care encounters requiring acute care utilization. The follow-up period spanned 1 year from the participant's survey date. The type of visit was confirmed in the electronic health record and medical claims as urgent, unplanned, or not elective. Each visit was further categorized as all-cause or potentially avoidable by a review of coded reasons for the visit/admission and a review of the medical chart notes, with the categorization guided by previously published criteria for categories of avoidable acute care visit types by the Centers for Medicare and Medicaid Services (CMS) quality indicators for patients with cancer (anemia, nausea, fever, dehydration, neutropenia, diarrhea, pain, pneumonia, sepsis, or emesis) (Alishahi Tabriz et al., 2023; Qualitynet, n.d.). For analyses, the outcome of acute care (AC) visits was categorized dichotomously (any AC visit vs. none during 1-year follow-up) or as the total number of AC visits during 1-year follow-up.

### Financial toxicity and other covariates

To assess FT, patients completed a survey including the ENRICh FT instrument, a measure comprised of 15 items for patient-reported severity of cancer-related financial burden (Smith et al., 2021). The global FT score, representing the overall FT burden, was calculated along with the scores for material FT and coping FT subdimensions. The global and subdimension scores range from 0 to 10, with 0 representing the least FT burden and 10 representing the most severe FT burden. The material FT subdimension score reflects financial depletion from aspects such as out-of-pocket medical costs, spent savings, accumulated debt, and lost income related to the respondent's cancer diagnosis, treatment, and survivorship. The coping FT subdimension score reflects the severity of depletion of resources to cope with FT burdens, such as savings or income, employment benefits, and formal organization-based resources (e.g., charities and professional organizations) and informal resources to financially cope (e.g., financial and resource help from family and friends) (Lentz et al., 2019) (Supplementary material). The ENRICh FT measure and the subdimensions it measures have been examined to be valid and reliable in previously published psychometric analyses (Smith et al., 2021; Xu et al., 2022) and predictive of adverse health outcomes (Maldonado et al., 2021; Corrigan et al., 2022). Prior published psychometric analyses were conducted for item reduction and evaluation of reliability with high internal consistency and demonstrated criterion validity and known-group validity. In descriptive statistics, FT scores were presented by quartiles, and in analytic models for this analysis, FT scores were tested as continuous variables, as per prior published analyses (Corrigan et al., 2022; Xu et al., 2022).

Sociodemographic and clinical covariates including patient age (at survey), sex, race, ethnicity, cancer histology type, cancer acuity, chemotherapy use, and extent of disease (local, regional, and distant guided by SEER overall staging approach) were abstracted from the electronic health record. For analyses, based on distributions, race and ethnicity were recategorized as a dichotomous variable as White non-Hispanic vs. others (combining non-White Hispanic or non-Hispanic plus another Hispanic ethnicity); the extent of cancer stage was dichotomized as distant vs. local or regional; and the cancer histology type was recategorized as higher acuity cancer disease site vs. lower acuity cancer disease site based on the empiric distribution of acute care visit counts by patients with that disease type above and below the median number of visits. Higher acuity utilization disease types included gastrointestinal, head and neck, and lung cancers. Lower acuity cancer sites included breast, prostate, leukemia, lymphoma, myeloma, gynecologic, central nervous system, skin, soft tissue, genitourinary, neuroendocrine, thymus, thyroid, and unknown primary cancers.

### Statistical analysis

Univariable associations between patient sociodemographic and clinical characteristics with the dichotomous outcome of any AC visits were tested using Pearson's chi-square test or Fisher's exact test for categorical variables and the Wilcoxon rank sum test for continuous variables. The likelihood ratio chi-square test in a logistic model was used to examine the unadjusted association between global FT and any AC visits.

The association between FT and repeated episodes of AC visits was then examined using Poisson regression models. We specified Poisson regression models with a log-link function to estimate the relative risk (RR) of acute care visits across the FT scoring scale (from 0 to 10), with the estimate reflecting the increase in risk per each 1-unit increase in the score. A parsimonious final model was selected to reduce collinearity and include *a priori* clinically relevant covariates (Corrigan et al., 2022). Analyses were conducted using SAS Enterprise Guide version 7.11 (Cary, NC). Statistical tests were two-sided with a *P*-value of < 0.05 considered statistically significant.

## Results

### Patient characteristics

Among all participants (*N* = 225), 42 patients (18.6%) utilized any AC visit within 1 year of follow-up, for a total of 84 AC visits. Visits were comprised of 54 (64.3%) inpatient admissions and 30 ED visits (35.7%). The most frequent causes for AC visits were cellulitis (*n* = 8), pneumonia (*n* = 5), pleural effusion (*n* = 5), fever of unknown origin (*n* = 4), and dehydration (*n* = 4). The most frequent causes for visits requiring inpatient admission were cellulitis (*n* = 7), pleural effusion (*n* = 3), and abdominal abscess (*n* = 3). A total of 24 (28.5%) visits were categorized as potentially avoidable. Patients with regional and distant diseases were more likely to require any AC visits. The most common cancer types requiring AC visits included gastrointestinal (26.2%), head and neck (19.1%), breast (16.7%), and lung (14.3%) cancers (Table 1).

**Table 1:** Univariable associations of patient characteristics with the outcome of any acute care (AC) Visits by 1-year follow-up.

	**Any AC visit *n* (%)**	**No AC visit *n* (%)**	***P*-value**
**Age**, median (interquartile range)	60.6 (52.0–70.1)	63.1 (53.6–70.1)	0.44
**Sex**			0.14
Female	19 (45.2%)	106 (57.9%)	
Male	23 (54.8%)	77 (42.1%)	
**Race and ethnicity**			0.91
White non-Hispanic	30 (71.4%)	138 (75.4%)	
Any Hispanic or Latino	7 (16.7%)	25 (13.7%)	
Black or African American Non-Hispanic	4 (9.5%)	15 (8.2%)	
Asian Non-Hispanic	1 (2.4%)	5 (2.7%)	
**Education**			0.90
Less than high school	1 (2.4%)	4 (2.2%)	
High school or GED	6 (14.3%)	37 (20.2%)	
Some college, associate degree or trade certification	17 (40.5%)	72 (39.3%)	
College degree (BS, BA)	10 (23.8%)	43 (23.5%)	
Graduate degree (MS, MA)	5 (11.9%)	18 (9.8%)	
Advanced degree (PhD, MD, JD)	3 (7.1%)	8 (4.4%)	
No response	0 (0.0%)	1 (0.6%)	
**Household income**			0.50
$0–$19,999	3 (7.3%)	16 (8.8%)	
$20,000–$49,999	11 (26.8%)	30 (16.6%)	
$50,000–$74,999	6 (14.6%)	32 (17.7%)	
$75,000 or more	21 (51.2%)	103 (56.9%)	
**Insurance**			1.00
Employer or marketplace-based	23 (54.7%)	99 (54.1%)	
Medicaid	1 (2.4%)	5 (2.7%)	
Medicare	17 (40.5%)	74 (40.4%)	
Other	1 (2.4%)	5 (2.7%)	
**Cancer type**			**< 0.001**
Breast	7 (16.7%)	75 (41.0%)	
Central nervous system	1 (2.4%)	3 (1.6%)	
Gastrointestinal	11 (26.2%)	13 (7.1%)	
Gynecological	3 (7.1%)	4 (2.2%)	
Head and neck	8 (19.1%)	18 (9.8%)	
Leukemia/lymphoma/myeloma	4 (9.5%)	11 (6.0%)	
Lung	6 (14.3%)	18 (9.8%)	
Prostate	0 (0.0%)	28 (15.3%)	
Other	2 (4.8%)	13 (7.1%)	
**Disease extent**			**0.001**
Local	7 (16.7%)	90 (49.2%)	
Regional	16 (38.1%)	50 (27.3%)	
Distant	15 (35.7%)	35 (19.1%)	

The bold values indicate *P* < 0.05.

### Severity of FT burden and subsequent risks of AC visits

**Figure 1:**
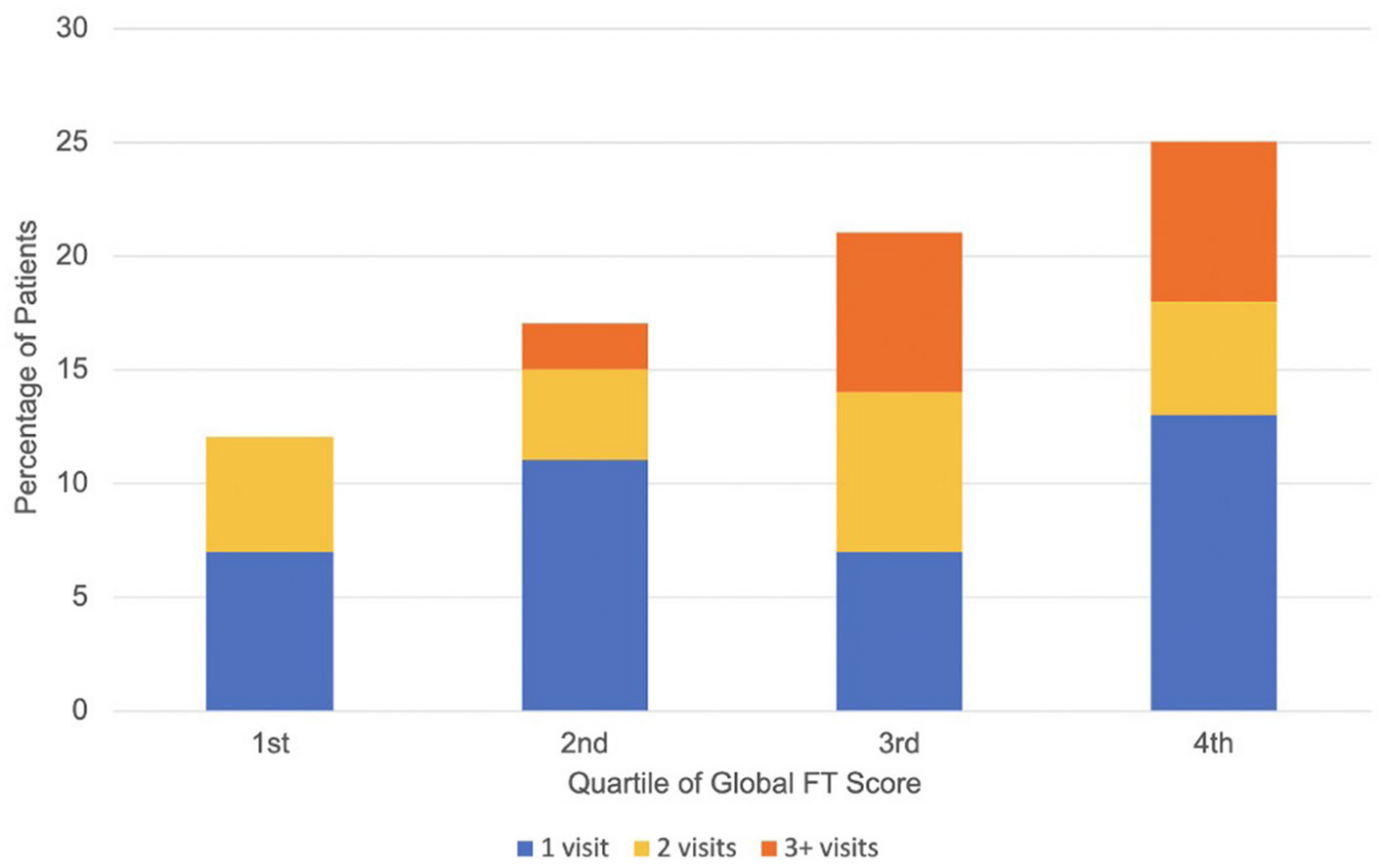
Distribution of acute care visit frequency by quartile of global financial toxicity score. Higher quartile represents worse financial toxicity.

The median time from survey respondents' diagnoses of cancer to their FT survey was 7.4 months (interquartile range 3.4–13.2). A total of 12.5% of patients with the lowest global FT burden (1st quartile of FT scores) required any AC visit within 1-year follow-up compared with 17.9% of patients in the second quartile, 21.1% of patients in the third quartile, and 25.0% of patients in the fourth quartile (most severe FT burden). The distribution of patients requiring multiple AC visits is also shown in Figure 1. A total of 7.0 and 7.1% of patients in the third and fourth quartile of FT burden had three or more AC visits, while 0.0 and 2.5% of patients in the first and second quartile had three or more visits. On unadjusted analysis, there was a trend toward significance in the association between global FT burden and the likelihood of any AC visit [Odds Ratio (OR) = 1.11; 95% Confidence Interval (CI) 0.99–1.24; *P* = 0.087 for every unit increase in the ENRICh FT score]. Global FT burden was associated with a lower likelihood of potentially avoidable (vs. all-cause) AC visit (OR = 0.74, 95% CI 0.85–0.96; *P* = **0.02**).

Global FT burden was associated with the risk of repeat AC visits within 1-year follow-up (RR = 1.17, 95% CI 1.07–1.29, ***P*<** **0.001** for every unit increase), even after adjusting for sociodemographic covariates, disease acuity type, and disease extent (Table 2). When examining subdimensions of FT, the burden of depleted FT coping resources (coping FT) was strongly associated with the risk of repeat AC visits (RR = 1.27, 95% CI 1.15–1.40, ***P*<** **0.001**), while material FT burden showed a trend toward association (RR = 1.07, 95% CI 0.99–1.15, *P* = 0.07) (Table 3). When examining the outcome of repeated potentially avoidable AC visits vs. all-cause visits or no visits, there was not a significant adjusted association with FT measures (Global FT RR = 0.96, 95% CI 0.80–1.16, *P* = 0.69; Material FT RR = 0.94, 95% CI 0.81–1.09, *P* = 0.43; Coping FT RR = 1.02 RR = 0.83–1.25, *P* = 0.84). The acuity of the disease site was associated with the risk of repeated AC visits in these models, while age was not significantly associated including in sensitivity analyses that characterized age categorically (Supplementary material).

**Table 2:** Multivariable predictors of repeated acute care visits by global financial toxicity (FT) score.

	**Global FT**		
	**Estimate**	**95% CI**	***P*** **-value**
**FT score**	1.17	1.07–1.29	**< 0.001**
**Age**	1.01	0.99–1.03	0.39
**Race/ethnicity**			
White non-Hispanic	1		
Other	1.48	0.91–2.38	0.11
**Cancer type**			
Higher acuity cancer disease site	1		
Lower acuity cancer disease site	3.22	2.05–5.05	**< 0.001**
**Disease extent**			
Local or regional	1		
Distant metastases	1.09	0.68–1.75	0.72

The bold values indicate *P* < 0.05.

**Table 3:** Multivariable predictors of repeated acute care visits by coping and material financial toxicity (FT) subdimension scores.

	**Material FT**			**Coping FT**		
	**Estimate**	**95% CI**	***P*** **-value**	**Estimate**	**95% CI**	***P*** **-value**
**FT score**	1.07	0.99–1.15	0.07	1.27	1.15–1.40	**< 0.001**
**Age**	1.00	0.99–1.02	0.66	1.01	0.99–1.03	0.22
**Race/ethnicity**						0.38
White non-Hispanic	1			1		
Others	1.55	0.95–2.51	0.08	1.44	0.90–2.33	0.13
**Cancer type**						
Higher acuity cancer disease site	1			1		
Lower acuity cancer disease site	3.11	1.99–4.88	**< 0.0001**	3.54	2.24–5.60	**< 0.001**
**Disease extent**						
Local or regional	1			1		
Distant metastases	1.15	0.71–1.85	0.57	1.06	0.66–1.70	0.80

## Discussion

In our study cohort of adult cancer patients with a spectrum of disease types undergoing comprehensive cancer care, individuals with the highest quartiles of severity of cancer-related FT at the study baseline showed significant, excess risks of subsequent acute oncology care utilization through 1-year follow-up. This included excess risk of all-cause and potentially avoidable clinical indications for care, with the vast majority of clinical encounters, more than 70%, not being potentially avoidable. More severe FT coping resource depletion—including the depletion of material, employment, professional, and social support resources—was especially predictive of subsequent repeat AC visits. Coping resource depletion was a stronger predictor of these repeat AC visits than direct material depletion. FT as a predictor of repeat AC visits remained significant even after accounting for disease type acuity and extent.

While consistent with evidence that lower socioeconomic status is associated with more frequent acute care use in general medical populations (Hong et al., 2007; Lash et al., 2017), results from the present study provide additional insight to our previously reported data specific to cancer patients, which identified that patients with more severe cancer-related FT baseline were more likely to miss routine oncology care visits (Maldonado et al., 2021) but accumulate excess unpaid medical debt within 6-month follow-up. Collectively with results from the prior study, the present analysis suggests a possible explanatory mechanism, where patients experiencing severe resource privations have a paucity of financial, coping, and social resources that contribute to lower access or adherence to planned, non-urgent oncology care visits in the short term. However, missed routine or necessary visits subsequently lead to higher risks of acute clinical complications and unmet supportive care needs, resulting in a higher frequency of urgent care use on longer-term follow-up (Hong et al., 2007). What remains needed in the additional prospective study is to determine whether this association is causal. Furthermore, future investigation is needed to discern whether efforts for early identification of and financial navigation in high-risk cancer patients with FT will translate into a meaningful decrease in acute oncology care resource burdens for healthcare systems (Raghavan et al., 2021), especially given the finding in our data that the acute visits in patients with severe FT were more likely all-cause than potentially avoidable.

There are limitations to consider. Though the patient sample had a variety of tumor types and acuity, this study was based at one comprehensive cancer center in a single metropolitan area in the USA, and therefore, additional studies to validate findings in highest-risk populations for FT, such as patients who are uninsured or receiving care through healthcare safety net systems, are still needed. Because the sample of survey respondents selected from this population of academic comprehensive cancer care center patients was comprised of 74.7% non-Hispanic White, 38.8% with a college degree or higher, 55.1% with an annual household income of $75,000 or more, and 97.3% with an insurance other than Medicaid public insurance, the results may have limited generalizability, particularly to uninsured and underinsured lower-income US populations. Another key issue is that the outcome of AC visits was defined by healthcare claims from care through the comprehensive cancer center and, therefore, focused on oncology care. Patients may have also sought acute care outside the hospital system, and these encounters were not captured.

## Conclusion

In this prospective study of acute oncology care utilization outcomes among adult cancer patients reporting a spectrum of financial burdens, FT measured using the validated multidimensional ENRICh tool was a predictor of a higher burden of acute care visits. The strongest association was demonstrated in patients reporting the most severely depleted FT coping resources (material, practical, and social resources), who subsequently were at risk for repeated ED visits and unplanned inpatient admissions. Findings emphasize the potential value of FT as a patient-reported outcome not only for predicting adverse downstream medical and economic outcomes seen in prior studies but also for predicting care delivery outcomes that impact individuals and healthcare systems. Future studies are needed to identify whether early FT screening and intervention efforts may help to mitigate urgent acute care utilization burdens.

## Data availability statement

The original contributions presented in the study are included in the article/Supplementary material, further inquiries can be directed to the corresponding author.

## Ethics statement

The studies involving humans were approved by MD Anderson Cancer Center Institutional Review Board. The studies were conducted in accordance with the local legislation and institutional requirements. The Ethics Committee/Institutional Review Board waived the requirement of written informed consent for participation from the participants or the participants' legal guardians/next of kin because it was a minimal risk, confidential electronic survey study where written informed consent would be burdensome on participants.

## Author contributions

SP, RV, SG, Y-CS, GS, and C-FW contributed significantly to the experimental design. JS, JM, SP, Y-SC, KK, and KD implemented the data. JS, JM, KD, SP, C-FW, Y-CS, and GS analyzed and interpreted the data. All authors contributed to the writing or revision and approval of the manuscript.
