# Supplementary material for: Financial toxicity in cancer patients and subsequent risk of repeat acute care utilization
Source: Front Psychol. 2023 Aug 17;14:1209526. doi: 10.3389/fpsyg.2023.1209526 (PMC10469858; doi:10.3389/fpsyg.2023.1209526)
Supplement: Supplementary file 1 [file Data_Sheet_1.docx]

Supplementary Material.

Economic Strain and Resilience in Cancer (ENRICh) financial toxicity instrument. Patients are asked to score items from 0 to 10.

Part 1: Please rate, during the past month, how has your disease and/or treatment affected: 1. Your spending on medical bills; 2. Money in your savings; 3. Other money you owe (like debts and credit cards); 4. Your ability to pay all of your bills; 5. Your ability to pay for food; 6. Your ability to work your usual number of hours at your job; 7. Your ability to contribute to your normal household responsibilities and daily chores; 8. Your stress level about finances; 0 = “not at all affected” to 10 = “affected a great deal”.

Part 2: During the past month, to deal with the financial impact of your disease and/or treatment, how much did you rely on: 1. Using your household income; 2. Using your savings; 3. Using credit cards; 4. Having someone to help manage your medical bills; 5. Having someone to help with your normal household responsibilities and daily chores; 6. Having someone to help care for the people who normally depend on you; 7. Having help from community resources (like churches, foundations, patient assistance, etc.); 0 = “did not rely at all” to 10= “relied a great deal”.

Sensitivity analysis #1: Poisson regression model, multivariable predictors of repeated acute care visits by financial toxicity (FT) scores when age is dichotomized by the median

|  | Global FT | | | Material FT | | | Coping FT | | |
| --- | --- | --- | --- | --- | --- | --- | --- | --- | --- |
|  | Estimate | 95% CI | P Value | Estimate | 95% CI | P Value | Estimate | 95% CI | P Value |
| **FT Score** | 1.18 | 1.07-1.29 | .001 | 1.07 | 1-1.15 | 0.061 | 1.27 | 1.15-1.4 | <.0001 |
| **Age** |  |  |  |  |  |  |  |  |  |
| 20-60 | 1 |  |  |  |  |  |  |  |  |
| 60+ | 1.33 | 0.84-2.11 | 0.221 | 1.24 | 0.78-1.97 | 0.364 | 1.41 | 0.89-2.23 | 0.139 |
| **Race/Ethnicity** |  |  |  |  |  |  |  |  |  |
| White Non-Hispanic | 1 |  |  |  |  |  |  |  |  |
| Other | 1.48 | 0.92-2.39 | 0.105 | 1.58 | 0.98-2.55 | 0.063 | 1.42 | 0.88-2.28 | 0.148 |
| **Cancer Type** |  |  |  |  |  |  |  |  |  |
| Lower acuity cancer disease site | 1 |  |  |  |  |  |  |  |  |
| Higher acuity cancer disease site | 3.24 | 2.06-5.07 | <.0001 | 3.12 | 1.99-4.88 | <.0001 | 3.58 | 2.27-5.66 | <.0001 |
| **Disease Extent** |  |  |  |  |  |  |  |  |  |
| Local or regional | 1 |  |  |  |  |  |  |  |  |
| Distant metastases | 1.08 | 0.67-1.74 | 0.741 | 1.13 | 0.7-1.82 | 0.612 | 1.06 | 0.66-1.7 | 0.799 |

Sensitivity Analysis #2: : Poisson regression model, multivariable predictors of repeated acute care visits by financial toxicity (FT) scores when age is categorized by quantile

|  | Global FT | | | Material FT | | | Coping FT | | |
| --- | --- | --- | --- | --- | --- | --- | --- | --- | --- |
|  | Estimate | 95% CI | P Value | Estimate | 95% CI | P Value | Estimate | 95% CI | P Value |
| **FT Score** | 1.17 | 1.07-1.29 | 0.001 | 1.07 | 0.99-1.15 | 0.083 | 1.27 | 1.15-1.4 | <.0001 |
| **Age** |  |  |  |  |  |  |  |  |  |
| 20-50 | 1 |  |  |  |  |  |  |  |  |
| 51-60 | 1.34 | 0.64-2.81 | 0.439 | 1.31 | 0.62-2.73 | 0.477 | 1.46 | 0.69-3.09 | 0.324 |
| 61-70 | 1.69 | 0.85-3.37 | 0.136 | 1.59 | 0.8-3.17 | 0.186 | 1.84 | 0.93-3.67 | 0.082 |
| 70+ | 1.44 | 0.66-3.12 | 0.361 | 1.25 | 0.58-2.71 | 0.567 | 1.66 | 0.76-3.63 | 0.205 |
| **Race/Ethnicity** |  |  |  |  |  |  |  |  |  |
| White Non-Hispanic | 1 |  |  |  |  |  |  |  |  |
| Other | 1.53 | 0.95-2.47 | 0.082 | 1.62 | 1-2.62 | 0.052 | 1.49 | 0.92-2.4 | 0.106 |
| **Cancer Type** |  |  |  |  |  |  |  |  |  |
| Lower acuity cancer disease site | 1 |  |  |  |  |  |  |  |  |
| Higher acuity cancer disease site | 3.14 | 2-4.95 | <.0001 | 3.05 | 1.94-4.8 | <.0001 | 3.44 | 2.17-5.46 | <.0001 |
| **Disease Extent** |  |  |  |  |  |  |  |  |  |
| Local or regional | 1 |  |  |  |  |  |  |  |  |
| Distant metastases | 1.07 | 0.67-1.73 | 0.767 | 1.12 | 0.7-1.81 | 0.639 | 1.05 | 0.65-1.68 | 0.842 |
